# Sac Shrinkage after Endovascular Aneurysm Repair: New Insights into Aneurysm Wall Degeneration and Loss of Elasticity

**DOI:** 10.5761/atcs.ra.25-00196

**Published:** 2026-02-13

**Authors:** Toshiya Nishibe, Tsuyoshi Iwasa, Masaki Kano, Akinari Iwahori, Jun Koizumi, Masayasu Nishibe, Alan Dardik

**Affiliations:** 1 Department of Medical Informatics and Management, Hokkaido Information University, Ebetsu, Hokkaido, Japan; 2 Department of Cardiovascular Surgery, Tokyo Medical University, Tokyo, Japan; 3 Department of Radiology, Chiba University School of Medicine, Chiba, Chiba, Japan; 4 Department of Surgery, Eniwa Midorino Clinic, Eniwa, Hokkaido, Japan; 5 Department of Vascular Surgery, Icahn School of Medicine at Mount Sinai, New York, USA

**Keywords:** abdominal aortic aneurysm, endovascular aneurysm repair, sac shrinkage, aneurysm wall degeneration, pulse wave velocity

## Abstract

Endovascular aneurysm repair (EVAR) has markedly reduced perioperative morbidity and mortality in the management of abdominal aortic aneurysms. However, its long-term durability remains a concern because of sac expansion, endoleaks, and late rupture. Sac shrinkage serves as a key surrogate marker of procedural success, reflecting favorable remodeling and a lower risk of complications. Sac behavior after EVAR is determined by the interplay between intrasac pressure and aneurysm wall integrity. Although pressure reduction is essential, wall degeneration and stiffness critically influence remodeling outcomes. The presence of simple renal cysts, a localized manifestation of systemic connective tissue degeneration, has been associated with impaired sac shrinkage and may indicate unfavorable remodeling. Arterial stiffness, assessed by pulse wave velocity, also correlates with sac behavior: lower stiffness favors shrinkage, whereas higher stiffness is linked to expansion or lack of shrinkage. These findings suggest that sac remodeling is a multifactorial process not solely dependent on flow exclusion. Future prospective studies integrating artificial intelligence, vascular remodeling inhibition, and stent graft innovation are warranted to refine patient-specific risk stratification, guide individualized surveillance, and promote sac shrinkage, thereby improving outcomes after EVAR.

## Introduction

Endovascular aneurysm repair (EVAR) has revolutionized the management of abdominal aortic aneurysms (AAAs) by providing a minimally invasive alternative to open repair. First introduced by Volodos in 1987 and clinically validated by Parodi in 1991,^1,2)^ EVAR is now established as a global standard of care. Its benefits, particularly lower perioperative morbidity and mortality, are most evident in elderly patients and those with substantial comorbidities.^3)^ However, concerns remain regarding its long-term durability. Compared with open repair, EVAR carries higher rates of secondary intervention and late rupture, often related to persistent sac pressurization, sac enlargement, or endoleak.^4)^

Among clinical markers, sac shrinkage has emerged as a key surrogate marker of procedural success, yet it is relatively uncommon, as shown in our cohort with rates of 15% and 18% at 1 and 2 years for polyester-based stent grafts and 30.3% and 37.5% for expanded polytetrafluoroethylene-based stent grafts.^5,6)^ Particularly, sac shrinkage is associated with fewer complications and a reduced need for surveillance, whereas stable or enlarging sacs signal a higher risk of rupture and reintervention.^7,8)^ Sac behavior after EVAR is largely associated with intrasac pressure, but patients with relatively high or low pressure do not consistently exhibit expansion or shrinkage.^9)^

A meta-analysis by Lalys et al. suggested that several clinical and anatomical factors, including renal impairment, type I and II endoleaks, hypercholesterolemia, smoking, statin therapy, coronary artery disease, diabetes, and intramural thrombus, may affect sac remodeling after EVAR.^10)^ Similarly, a systematic review by van Rijswijk et al. reported that aneurysm anatomy such as neck or AAA thrombus, hostile neck parameters, AAA volumetric parameters, and aortic calcification may also play a role,^11)^ but overall the evidence remains inconclusive.

Recently, aneurysm wall properties such as degeneration and elasticity have attracted considerable attention as one of the key determinants of sac behavior.^12,13)^ Sac behavior is possibly determined by the balance between intrasac pressure and wall properties, which are heterogeneous: some walls exhibit elasticity, whereas others lack it, potentially influencing sac shrinkage. These heterogeneous properties also reflect risk factors previously reported in the literature, such as atherosclerosis, genetic predisposition, intraluminal thrombus, inflammation, and aneurysm morphology.^10,13)^

**Fig. 1:**
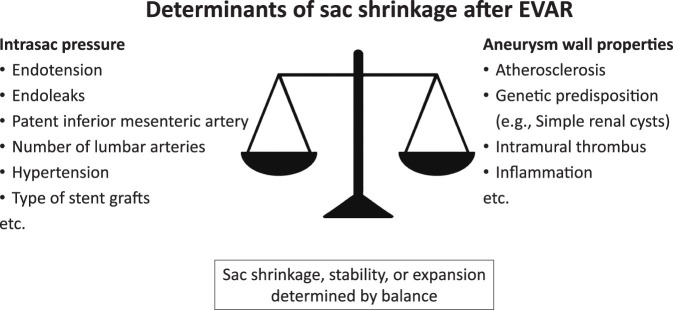
Conceptual balance model illustrating factors influencing sac shrinkage after EVAR. Promoting factors, such as aneurysm wall properties, are balanced against limiting factors, such as intrasac pressure. Intrasac pressure is influenced by endotension, endoleaks, a patent inferior mesenteric artery, the number of lumbar arteries, hypertension, and the type of stent graft, while aneurysm wall properties are affected by atherosclerosis, genetic predisposition (e.g., simple renal cysts), intramural thrombus, and inflammation. EVAR: endovascular aneurysm repair

Sac remodeling is now recognized as a multifactorial process influenced not only by clinical and anatomical factors or endoleaks^9)^ but also by the properties of the aneurysm wall, such as aneurysm wall degeneration and elasticity, which have emerged as a potentially important determinant of sac behavior (**Fig. 1**). This review summarizes current insights into the mechanisms underlying sac shrinkage after EVAR, with a particular focus on aneurysm wall degeneration and loss of elasticity to improve long-term outcomes and guide postoperative management strategies.

## Intrasac Pressure and Sac Behavior

EVAR aims to completely exclude blood flow from the aneurysm to promote sac shrinkage, with intrasac pressure or endotension serving as a central determinant of sac behavior. This pressure, influenced by device-related factors (e.g., endoleak, graft porosity, compliance) and anatomical features (e.g., side branch patency, sac morphology, thrombus characteristics), does not fall to zero and varies between cases. Reported post-EVAR mean pressures range from 16 to 92 mmHg, and pulse pressures from 1 to 47 mmHg,^14–17)^ consistent with our current data showing averages of 59.6 and 19.5 mmHg, respectively.^12)^ Sac behaviors are known to be closely linked to intrasac pressure; low pressure favors shrinkage and high pressure favors expansion, whereas intermediate pressure yields inconsistent sac remodeling.^9)^ Computational simulations suggest approximately 60 mmHg as a critical threshold for sac stability.^18)^ Our clinical study showed that at this level, intrasac pressure remained within a range where sac shrinkage or expansion could not be reliably predicted.^19)^

## Aneurysm Wall Degeneration and Sac Remodeling after EVAR

### Possible role of aneurysm wall degeneration in sac remodeling after EVAR

**Fig. 2:**
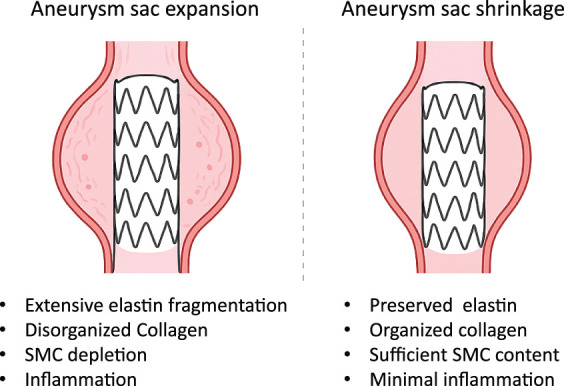
Role of structural integrity and elasticity in sac remodeling after EVAR. Aneurysm walls with preserved elastin, organized collagen, sufficient SMC content, and minimal inflammation tend to undergo sac shrinkage, whereas degenerated walls with extensive elastin fragmentation, disorganized collagen, SMC depletion, and inflammation are prone to sac expansion despite reduced intrasac pressure. EVAR: endovascular aneurysm repair; SMC: smooth muscle cell

The aneurysm wall undergoes marked structural and biochemical remodeling characterized by elastin degradation, collagen disorganization, and smooth muscle cell depletion. Upregulation of matrix metalloproteinases (MMP-2, MMP-9, MMP-12) accelerates extracellular matrix breakdown, resulting in progressive weakening of the vessel wall.^20)^ These pathological changes are further influenced by atherosclerosis, intramural thrombus, and inflammation, which collectively contribute to sac expansion.^13)^ Such degenerative changes are considered to impair sac remodeling under residual pressure after EVAR. Preservation of structural integrity and elasticity can promote sac shrinkage after EVAR, whereas severely degenerated or fibrotic aneurysm walls may show only limited remodeling even under reduced intrasac pressure (**Fig. 2**).^18)^ Adaptive remodeling allows sac shrinkage under reduced hemodynamic load through the maintenance of wall compliance, but stiff, fibrotic walls lack this adaptive potential.

### Simple renal cysts and post-EVAR sac shrinkage

Simple renal cysts (SRCs) are considered to be a localized manifestation of systemic connective tissue degeneration.^21)^ Recent studies have demonstrated an increased prevalence of AAAs in patients with SRCs, suggesting that AAAs and SRCs may share common pathophysiologic mechanisms.^22–26)^ Two meta-analyses reported that SRCs were 2.5- and 2.61-fold more prevalent in patients with AAAs than in those without AAAs.^27,28)^ Degradation of extracellular matrix components, such as elastin and collagen, by MMPs plays a crucial role in the development of AAAs as well as SRCs.^21)^ Furthermore, genetic polymorphisms in MMP-related genes have been associated with increased susceptibility to both AAAs and SRC formation.^29)^

**Fig. 3:**
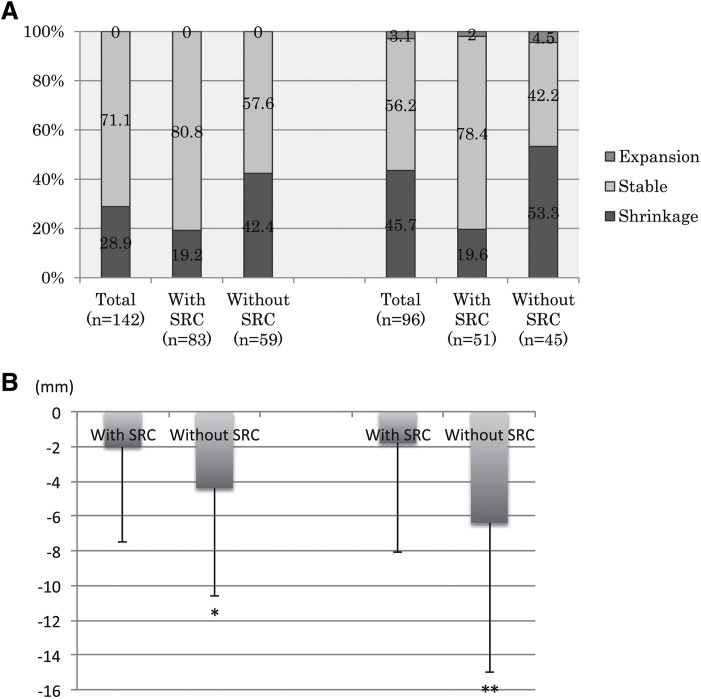
Aneurysm sac shrinkage after EVAR according to the presence or absence of SRCs. *P = 0.002; **P = 0.005. (**A**) Percentages of aneurysm sac shrinkage, stable aneurysm sac, and expansion of aneurysm sac at 1 year (left panel) and 2 years (right panel) after EVAR. Shrinkage was defined as a ≥5-mm decrease in the maximum short-axis diameter of the aneurysm sac compared with baseline; expansion as a ≥5-mm increase; and stable as a change <5 mm. (**B**) Average sac reduction compared with baseline at 1 year (left panel) and 2 years (right panel). EVAR: endovascular aneurysm repair; SRCs: simple renal cysts. Reproduced from Nishibe et al.^12)^ ©2019 Elsevier Inc.

Our study, which included 155 patients undergoing EVAR, demonstrated the presence of SRCs in 59%.^12)^ Patients with SRCs exhibited significantly lower rates of sac shrinkage at 1 and 2 years compared with those without SRCs (19.2% vs. 42.4% [P = 0.03] and 19.6% vs. 53.3% [P = 0.01], respectively; **Fig. 3A**). Patients with SRCs showed significantly less sac shrinkage than those without SRCs at 1 year and 2 years (2.0 ± 5.5 mm vs. 4.4 ± 6.2 mm [P = 0.002] and 1.8 ± 6.3 mm vs. 6.4 ± 8.6 mm [P = 0.005], respectively; **Fig. 3B**). Multivariable analysis identified SRCs (odds ratio [OR], 0.28; 95% confidence interval [CI], 0.12–0.63; P = 0.002) as an independent predictor of impaired sac remodeling. These results, showing impaired sac shrinkage after EVAR in AAAs with SRCs, support the hypothesis that aneurysms with structurally degenerated walls may exhibit limited remodeling capacity, even under reduced intrasac pressure. There were no significant differences in baseline characteristics or clinical and anatomical risk factors, except sex and chronic kidney disease, between AAAs with and without SRCs.

## Arterial Stiffness and Sac Remodeling after EVAR

### Arterial stiffness and sac shrinkage after EVAR

Arterial stiffness reflects a loss of arterial elasticity, which arises from structural degradation of elastin and collagen within the aortic wall, particularly in the medial and adventitial layers. These pathophysiological changes, accelerated by aging, chronic inflammation, and mechanical fatigue, reduce vascular compliance and impair the wall’s capacity for adaptive remodeling. Pulse wave velocity (PWV) can provide a reproducible, noninvasive measure of arterial stiffness.^30)^ PWV is closely related to atherosclerosis, intramural thrombus, and inflammation, all of which influence arterial wall properties.^30,31)^ PWV is also well established as a surrogate marker of vascular aging and a predictor of adverse cardiovascular outcomes. Recent evidence also links elevated PWV to the development and progression of AAAs, highlighting its significance for both preoperative risk assessment and postoperative surveillance.^32)^

Several studies have identified preoperative PWV, along with aneurysm diameter and type II endoleak, as independent risk factors associated with aneurysm sac shrinkage (**Table 1**).^33–35)^ Hori et al. demonstrated that lower PWV was a significant predictor of sac shrinkage at 3 years post-EVAR (OR, 0.32; P = 0.022).^33)^ We also reported that patients with sac shrinkage had significantly lower PWV values than those without shrinkage. Receiver operating characteristic (ROC) analysis identified 17.79 m/s as the optimal cutoff, with patients below this threshold being roughly 4 times more likely to achieve sac shrinkage (OR, 0.25; 95% CI, 0.11–0.57; P <0.001).^34)^ Similarly, Ugajin et al. found that PWV above 18.50 m/s was associated with increased risk of sac expansion (OR 3.059; P = 0.005).^35)^ These findings indicate that aneurysm wall degeneration, reflected by increased arterial stiffness, impairs sac shrinkage after EVAR, likely due to medial dysfunction and loss of elastic recoil.

**Table 1:** List of published studies on pulse wave velocity (PWV) and sac behavior after endovascular aneurysm repair (EVAR)

Author (year)	Study design	Sample size	PWV cutoff (m/s)	Outcome	OR/HR	95% CI	P-value
Preoperative PWV							
Hori et al. (2019)^33)^	Retrospective, single center	135	N/A	Sac shrinkage at 3 years	OR 0.32	0.12–0.84	0.022
Nishibe et al. (2021)^34)^	Retrospective, single center	119	17.79	Sac shrinkage at 2 years	OR 0.25	0.11–0.57	<0.001
Ugajin et al. (2022)^35)^	Retrospective, single center	175	18.54	Sac growth	HR 3.059	1.41–6.64	0.005
Postoperative PWV							
Hori et al. (2019)^33)^	Retrospective, single center	135	N/A	Sac expansion at 3 years	OR 3.80	1.02–10.70	0.048

OR: odds ratio; HR: hazard ratio; CI: confidence interval; N/A: not available

Hori et al. also reported that postoperative PWV (OR, 3.80; 95% CI, 1.02–10.70; P = 0.047) was independently associated with sac expansion.^33)^ Such postoperative increase in PWV appears to elevate intrasac pulse pressure, indirectly affecting sac behavior by retrograde or collateral transmission of pulsatile forces, consistent with type I or II endoleak.

### Mechanistic relationship between PWV and aneurysm wall elasticity

**Fig. 4:**
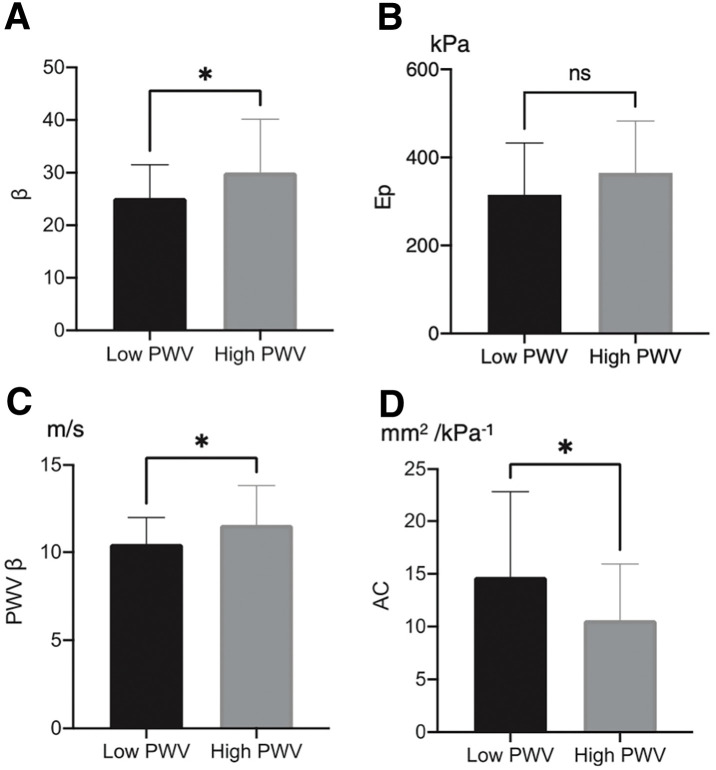
Comparison of regional arterial stiffness and distensibility parameters between patients with high and low PWV. High PWV was defined as ≥1800 cm/s and low PWV as <1800 cm/s. *Significant; NS, not significant. (**A**) Stiffness parameter (β), (**B**) pressure–strain elasticity modulus (Ep), (**C**) 1-point pulse wave velocity (PWV β), and (**D**) arterial compliance. PWV: pulse wave velocity. Reproduced from Nishibe et al.^36)^ under the terms of the Creative Commons Attribution License (CC BY).

Recent progress in imaging techniques and physiological measurements allows for direct characterization of aneurysm sac wall properties. Our study of 49 patients demonstrated that those with elevated brachial–ankle PWV (≥1800 cm/s) had significantly higher local wall stiffness, reflected by stiffness parameter β (30.6 ± 10.1 vs. 25.2 ± 6.3, P = 0.047) and 1-point PWV β (11.6 ± 2.3 vs. 10.5 ± 1.5 m/s, P = 0.048), and significantly lower arterial compliance (10.6 ± 5.3 vs. 14.7 ± 8.1 mm^2^/kPa^−1^, P = 0.045) compared to those with lower PWV (**Fig. 4**).^36)^ Arterial compliance also showed a significant negative correlation with PWV (r = −0.361, P = 0.011), suggesting that reduced distensibility reflects increased mechanical stress.

These results align with magnetic resonance imaging–based 4-dimensional flow analyses by Lindenberger et al. showing elevated global and abdominal aortic PWV in AAA patients (global PWV: 8.9 ± 2.4 m/s vs. 7.1 ± 1.5 m/s in controls, P = 0.007; abdominal PWV: 7.0 ± 1.8 m/s vs. 5.8 ± 1.0 m/s, P = 0.022), while descending thoracic PWV remained unchanged (8.7 ± 3.2 m/s vs. 8.2 ± 2.4 m/s, P = 0.59).^37)^ These findings support that arterial wall stiffening is not uniformly distributed but is preferentially concentrated at the site of aneurysmal degeneration.

## Link between SRCs and Arterial Stiffness in AAA

Our subsequent study involving 223 patients with AAAs further demonstrated that SRC is independently associated with increased arterial stiffness.^38)^ Using brachial–ankle PWV, patients were classified into high (≥1800 cm/s) and normal/borderline groups. Increased stiffness was present in 60% of patients. Multivariable analysis identified age ≥75 years (OR, 2.83; 95% CI, 1.51–5.72; P = 0.002), systolic blood pressure ≥140 mmHg (OR, 5.05; 95% CI, 2.35–10.83; P <0.001), hypertension (OR, 2.28; 95% CI, 1.08–4.79; P = 0.030), and the presence of SRCs (OR, 1.89; 95% CI, 1.03–3.46; P = 0.040) as independent risk factors for increased arterial stiffness. This association suggests that SRCs are not an incidental finding but a marker of systemic connective tissue degeneration, closely linked to arterial stiffness and aneurysm wall fragility. SRC and PWV serve as interrelated biomarkers delineating a specific subgroup of AAA patients with impaired sac remodeling capacity.

## Clinical Implications and Future Perspectives

Predicting sac shrinkage after EVAR has important clinical implications; it enables refined risk stratification, identifies patients who are not suitable for the procedure, and guides tailored surveillance and timely adjunctive therapy, ultimately promoting shrinkage and improving outcomes.

### Artificial intelligence and machine learning

**Fig. 5:**
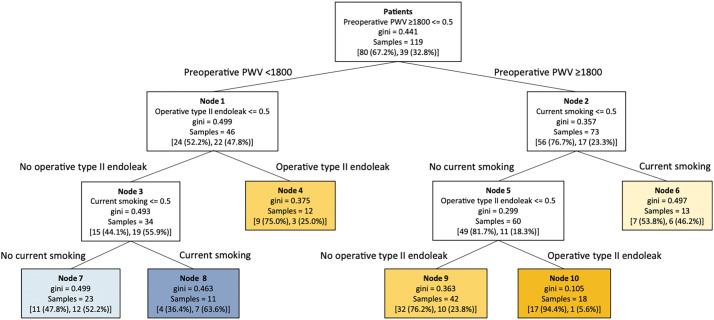
A decision tree illustrating the hierarchical association of predictors with aneurysm sac shrinkage. Preoperative PWV was the strongest predictor, followed by operative type II endoleak and current smoking. Each internal or terminal node shows the number and percentage of patients without sac shrinkage (left) and with sac shrinkage (right). For example, Node 8 (PWV <1800 cm/s, no type II endoleak, current smoking) shows the highest likelihood of sac shrinkage, with 63.6% of patients exhibiting shrinkage, whereas Node 10 (PWV ≥1800 cm/s, no current smoking, type II endoleak) shows the lowest likelihood, with only 5.6% exhibiting shrinkage. Blue nodes indicate a higher likelihood of sac shrinkage, and orange nodes indicate a lower likelihood. PWV: pulse wave velocity. Reproduced from Nishibe et al.^39)^ ©2024 Elsevier Inc.

The integration of diverse risk factors through artificial intelligence (AI) and machine learning holds promise for improving post-EVAR risk stratification, providing a multidimensional framework that may enhance predictions of sac behavior and support more tailored surveillance and intervention strategies. We are using machine learning to investigate AAA patients undergoing EVAR and to identify key predictors of aneurysm sac shrinkage.^39)^ To facilitate clinical stratification, we have developed a machine learning-based decision tree model incorporating these factors to estimate the likelihood of sac shrinkage. As shown in **Fig. 5**, preoperative PWV (<1800 or ≥1800 cm/s) is located at the uppermost hierarchical level of the decision tree, indicating its role as the strongest predictor of sac shrinkage. Type II endoleak and current smoking, which have been consistently reported as risk factors for impaired sac shrinkage,^10)^ are positioned at lower tiers of the tree. These findings emphasize that although type II endoleak and smoking remain clinically important, arterial stiffness reflected by increased PWV is the most critical determinant of sac behavior. Analyses using AI and machine learning can also uncover complex relationships among risk factors that conventional statistical approaches may not capture.

### Pharmacological intervention

Preserving wall integrity offers a promising strategy to promote sac regression after EVAR; however, current approaches remain preliminary, and further data are needed to establish their clinical relevance. Medications known to reduce PWV,^40)^ such as angiotensin-converting enzyme inhibitors (e.g., perindopril, enalapril), angiotensin II receptor blockers (e.g., losartan, valsartan), calcium channel blockers (e.g., amlodipine), statins (e.g., atorvastatin, rosuvastatin), and sodium glucose cotransporter 2 inhibitors and glucagon-like peptide-1 receptor agonists (e.g., canagliflozin, semaglutide) may enhance sac shrinkage not only through reduced blood pressure but also via anti-inflammatory effects and inhibition of vascular remodeling. Statin therapy has been reported to increase the likelihood of sac shrinkage following EVAR.^10,41)^ Careful appraisal of novel pharmacologic interventions targeting arterial stiffness is therefore warranted.

### Stent graft technology

Given the small number of reported cases, a previous study has suggested that differences in stent graft design, such as the Gore Excluder (W. L. Gore & Associates, Flagstaff, AZ, USA) and TREO systems (Terumo Corporation, Tokyo, Japan), may influence sac shrinkage by altering the transmission of pulsatile blood pressure into the aneurysm sac.^42)^ These observations suggest the potential role of device architecture in determining sac behavior.

## Conclusions

Sac shrinkage after EVAR is a key surrogate marker of procedural success, although its manifestation is modulated by a multifactorial interaction of mechanical, biological, and clinical factors. Sac remodeling is a multifactorial process influenced not only by clinical factors, anatomical factors, or endoleaks but also, by intrasac pressure and aneurysm wall properties. Current evidence suggests that patients with more degenerated or stiffened arterial walls are less likely to experience sac shrinkage, even when complete flow exclusion is achieved. Future prospective studies integrating AI, vascular remodeling inhibition, and stent graft innovation are warranted to refine patient-specific risk stratification, inform personalized postoperative management, and promote sac shrinkage, thereby improving outcomes after EVAR.
